# Happiness, quality of working life, and job satisfaction among nurses working in emergency departments in Iran

**DOI:** 10.1186/s12955-021-01755-3

**Published:** 2021-04-01

**Authors:** Somayeh Javanmardnejad, Razieh Bandari, Majideh Heravi-Karimooi, Nahid Rejeh, Hamid Sharif Nia, Ali Montazeri

**Affiliations:** 1 Faculty of Nursing and Midwifery, Shahed University, Tehran, Iran; 2 Social Determinants of Health Research Center, Semnan University of Medical Sciences, Semnan, Iran; 3 Elderly Care Research Center, Faculty of Nursing and Midwifery, Shahed University, Tehran, Iran; 4 School of Nursing and Midwifery Amol, Mazandaran University of Medical Sciences, Sari, Iran; 5 Population Health Research Group, Health Metrics Research Centre, Iranian Institute for Health Sciences Research, ACECR, Tehran, Iran; 6 Faculty of Humanity Sciences, University of Science and Culture, Tehran, Iran

**Keywords:** Quality of Work Life Questionnaire, Job Satisfaction, Happiness, Nurses, Emergency Department

## Abstract

**Background:**

Nurses have a vital role in the healthcare system. One of the basic steps to increase their happiness is to recognize factors such as job satisfaction and quality of working life. Therefore, the goal of the present study was to examine the relationship between happiness and quality of working life and job satisfaction among nursing personnel.

**Methods:**

This descriptive study was carried out on 270 hospital nurses who worked in emergency departments in Iran. Nurses were recruited through the census method. Data collection instruments included the Oxford Happiness Inventory (OHI), the Quality of Work Life Questionnaire (QWL), and the Job Satisfaction Questionnaire (JSQ). Data were explored using descriptive statistics, and stepwise multiple linear regression analysis.

**Results:**

The mean age of participants was 30.1 ± 6.26 years. The mean happiness score was 38.5 ± 16.22, the mean Quality of Working Life (QWL) score was 84.3 ± 17.62, and the mean job satisfaction score was found to be 45.5 ± 13.57); corresponding to moderate levels of attributes. The results obtained from the ordinary least-square (OLS) regression indicated that happiness significantly was associated with economic status and satisfaction with closure (R^2^: 0.38).

**Conclusion:**

Overall the current study found that nurses who work in emergency departments did not feel happy. Additionally, the findings suggest that their happiness were associated with their economic status, and closure over their duties.

## Background

Happiness is a positive feeling that is vital and significant to maintain health [1]. Even though the pursuit of happiness is as old as human history, research on the concept of happiness is relatively new [2]. Happiness is of great importance to all professions, particularly the nursing profession [3] because nurses are in direct and constant contact with patients and clients whose unique conditions require nurses to be altruistic, self-confident, dedicated, creative, kind, and energetic. All of these attributes are directly linked with happiness [4]. On the other hand, daily exposure to patients' pain and suffering, heavy workload, and poor working conditions impose high levels of occupational stress for nurses [5]. Occupational stress, in turn, undermines nurses' self-confidence and concentration, increases their irritability, brings them to sleep disorders and job burnout, and thereby, negatively affects their happiness and care quality [5, 6].

Nurses’ happiness becomes important because happiness or unhappiness may affect nurses’ abilities to help patients [3]. Because nurses have responsibilities to holistically care for sick, wounded, traumatized, and weak patients in their charge, they may be prone to experience negative feelings while performing their work [7, 8]. In addition, nurses often work in difficult circumstances, which include patient-related problems, heavy and intense workloads, staff shortages, an aging nursing workforce, ineffective policies that support nurses in administrative systems, inadequate supervisor support, unfair pay, poor working conditions, lack of resources and equipment to work effectively, limited career opportunities, limited educational opportunities, and unstable work environments. These difficult circumstances can have a daily impact on nurses’ emotional well-being and their ability to provide care [3].

Factors that influence happiness among nurses include positive and negative emotions, life satisfaction, personal and work-related dynamics. Of these, it has been shown that physical health status and the reasons behind entering nursing, friendly relationships, satisfaction with salary, workload, quality of life, the amount of clinical work experience, satisfaction with staff number in each shift, satisfaction with patients' and family members' feedbacks, satisfaction with the conduct and the performance of physicians, satisfaction with the conduct and the performance of colleagues, satisfaction with the conduct and the performance of the head-nurse, satisfaction with the conduct and the performance of hospital nursing office authorities, satisfaction with welfare facilities at workplace and job satisfaction are essential [3, 9, 10].

Because healthcare systems are among the largest service providers in the society [11], the improvement of the quality of working life of nursing personnel is an important factor in ensuring stability in the healthcare system. [12]. An optimum level of quality of working life enables nurses to provide high-quality services to patients, and this is only possible if they have proper good mental health, job satisfaction, and satisfaction with different areas of life. Therefore, the quality of life of a nurse both as a human being and as a person who takes care of other members of the society warrants special attention [13].

A good level of quality of working life for nurses is realized when they can satisfy their needs through working in the healthcare system and at the same time be able to achieve organizational objectives [14]. The quality of working life not only affects job satisfaction, but also influences other aspects of life, including family, and social relationships [15]. Therefore, it is argued that job satisfaction is a very important part of a nurse’s life, that influences her or his level of performance, early retirement, job transfers, organizational commitment, and also patient safety, and most importantly patient satisfaction [16]. Job satisfaction is an essential predictor of absence from work, occupational burnout, quitting the nursing profession, or intention to do so among nurses [17]. One of the essential steps in increasing productivity is to understand factors that are involved in job satisfaction, quality of life, and happiness of the nurses [18].

The emergency department is considered the heart of a hospital and has an essential status in the medical system due to the importance of early, high-quality, and effective care and because of the complex processes occurring in it. Emergency department nurses are faced with various problems that can affect their quality of working life, job satisfaction, and happiness. The present study aimed to explore the association between quality of working life and job satisfaction with happiness among emergency department nurses in Iran.

## Methods

### Design

This was a cross-sectional study conducted among emergency department nurses in hospitals affiliated to Ilam University of Medical Sciences, Ilam, Iran, in 2018. The study aimed to investigate happiness, quality of working life, and job satisfaction.

### Participants

All nurses working in emergency departments from all hospitals (n = 10) formed the study sample. However, the following inclusion and exclusion criteria were considered:

Inclusion criteria: at least have 6 months of experience working in an emergency department, and willingness to participate in the study. Exclusion criteria: working in other departments, and not willing to participate in the study.

### Procedure

The main investigator (SJ) attended all emergency departments on several occasions so that all nurses working in different shifts. The data collection was carried out at the beginning, middle, and end of each shift The data were collected using a number of self-reported questionnaires. Additionally, the demographic characteristics of participants including age, gender, marital status, employment status, work experience, monthly salary, and work shifts also were recorded. Incomplete questionnaires were excluded from the analysis.

### Measures

#### The Oxford happiness inventory (OHI)

It contains 29 items and seven subscales, satisfaction with life, efficacy, sociability/empathy, positive outlook, well-being, cheerfulness, and self-esteem. Each item is rated on a 4-point scale ranging from 0 to 3 (‘I do not feel happy’ to ‘I am incredibly happy’). The total score ranges from 0 to 87 with a higher score indicating greater happiness [19–21]. The validity and reliability of the Persian OHI were verified in previous studies and its Cronbach's alpha was reported to be 0.98 [22].

#### The quality of work life questionnaire (QWL)

It has 35 items covering eight subscales: adequate and fair compensation (four items), safe and healthy working conditions (six items), the opportunity to use and develop human capacities (five items), opportunity to growth and security (four items), social integration in the work organization (four items), the constitution in the work organization (four items), work and total life span (three items), and social relevance of work life (five items) [23]. The items are rated on a five-point Likert-scale ranging from 1 (very dissatisfied) to 5 (very satisfied). The score on the QWL ranges from 175 (highest) to 35 (lowest) [24]. Walton reported reliability of 0.88 for the questionnaire. In Iran, acceptable values for Cronbach’s alpha were reported (ranging from 0.83 to 0.91) [25].

#### The job satisfaction questionnaire (JSQ)

It contains 14 items are rated on a Likert-scale from 1 (strongly disagree) to 5 (strongly agree). The JSQ contains four subscales that include satisfaction with information, satisfaction with variety, satisfaction with closure, and satisfaction with pay. A higher total score indicates the member has higher job satisfaction. Sample items in JSQ are as follows: *I am satisfied with the information I receive from my superior about my job performance* (satisfaction with information), *I am satisfied with the variety of activities my job offer* (satisfaction with variety), *I am satisfied with the opportunities my job gives me to complete the tasks from beginning to end* (satisfaction with closure; the opportunity to complete working tasks), and *I am satisfied with the pay I receive for my job* (satisfaction with pay). [26]. The JSQ has been validated in Iran and Cronbach’s alpha value of 0.85 for the questionnaire was reported [27]. In the present study, the reliability of the JSQ was assessed using the Cronbach’s alpha to make sure about its reliability. We found an alpha value of 0.92 that indicated good reliability for the questionnaire**.**

### Statistical analysis

Data analyses were carried out using the SPSS software (v. 16.0) (SPSS Inc., Chicago, Illinois). The Kolmogorov–Smirnov test was used for normality assessment. Then, data analysis was performed in two steps. In the first step, descriptive statistics including frequency, mean, and standard deviation were used to explore the data. We also performed a correlation between happiness and all other variables to assess if a significant correlation exists. Consequently, in the second step, ordinary least square linear regression analysis was used to assess the relationship between happiness (dependent variable) and independent variables. In addition, collinearity diagnostics were reported. As such, we assumed if tolerance was between 0 to 1 and variance inflation factors (VIF) for each independent variable was less than 10 there were no concerns for multicollinearity [28]. All significant findings from correlation analyses were entered into the regression model. The level of significance in all analyses was set at less than 0.05.

## Results

### Characteristics of the study sample

In all, there were 285 nurses working in emergency departments. Of these, 270 nurses agreed to participate in the study giving a response rate of 95%. The remaining 15 nurses were excluded based on exclusion criteria. The mean age of participants was 30.12 ± 6.26 years and the mean work experience of nurses was 1.23 ± 0.51 years. The majority of participants were female (57.4%), 50.4% were married, and 84.8% had a bachelor degree. In addition, 76.7% worked in rotating shifts (Table 1).

### Happiness, quality of work life and job satisfaction: descriptive findings

The mean happiness score was 38.5 (SD = 16.22) and this for the Quality of Working Life (QWL) was 84.3 (SD = 17.62). In addition, the mean score for job satisfaction was found to be 45.5 (SD = 13.57). Overall the findings showed a moderate level of happiness, quality of working life, and job satisfaction for the study sample. The detailed results are presented in Table 2.

### Happiness, quality of work life and job satisfaction: correlations

The correlation between happiness, demographic information, quality of work life, and job satisfaction are presented in Tables 3, 4, 5, respectively. The findings showed that economic status, the Quality of Working Life (QWL) subscales, and the Job Satisfaction Questionnaire (JSQ) subscales were significantly correlated with overall happiness (P < 0.05).

**Table 1:** The characteristics of study participants (n = 270)

	Number (%)
*Gender*	
Female	155(57.4)
Male	115(42.6)
*Marital status*	
Married	136(50.4)
Single	134(49.6)
*Age group*	
22–32	191(70.7)
33–43	64(23.7)
44–55	15(5.6)
*Education*	
Practical nurse	19(7.0)
Bachelor degree	229(84.8)
Master degree	22(8.1)
*Economic status*	
Poor	58(21.5)
Intermediate	203(75.2)
Good	9(3.3)
*Work shift*	
Morning shift	38(14.1)
Evening shift	5(1.9)
Night shift	20(7.4)
Rotation shift	207(76.7)
*Employment status*	
Fixed term	201(74.4)
Permanent	69(25.6)
*Work experience*	
1–10 years	217(80.4)
11–20 years	42(15.6)
20–30 years	11(4.1)

**Table 2:** Happiness, quality of work life and job satisfaction among emergency department nurses

	Mean (SD)
*Happiness*	
Satisfaction with life	8.1(3.66)
Efficacy	17.9(4.49)
Sociability/empathy	8.1(3.47)
Positive outlook	7.5(3.75)
Well-being	3.4(1.97)
Cheerfulness	4.4(2.02)
Self-esteem	2.5(1.41)
*Total *	38.5(16.22)
*Quality of Work Life*	
Adequate and fair compensation	8.2(3.17)
Safe and healthy environment	14.1(3.93)
Development of human capacities	13.3(3.59)
Growth and security	9.5(2.82)
Social integration	10.4(3.19)
Constitutionalism	9.5(3.17)
Total life space	6.9(2.51)
Social relevance	12.1(3.97)
*Total*	84.3(17.62)
*Job Satisfaction*	
Satisfaction with Information	15.4(5.49)
Satisfaction with Variety	20.0(6.52)
Satisfaction with Closure	5.6(2.96)
Satisfaction with Pay	4.3(3.16)
*Total*	45.5(13.57)

**Table 3:** Correlation between happiness and demographic variables

	Total OHI	Age	Gender	Marital status	Education	Employment status	Economic status	Work experience	Work shift
Total OHI	1								
Age	− 0.068	1							
Gender	0.047	0.219^**^	1						
Marital status	− 0.077	− 0.429^**^	− 0.106	1					
Education	0.048	− 0.213^**^	0.014	0.086	1				
Employment status	− 0.069	0.686^**^	0.165^**^	− 0.429^**^	− 0.039	1			
Economic status	0.138^*^	− 0.001	0.018	0.027	0.091	0.027	1		
Work experience	− 0.039	0.830^**^	0.201^**^	− 0.329^**^	− 0.347^**^	0.574^**^	0.006	1	
Work shift	− 0.066	− 0.287^**^	− 0.054	0.143^*^	0.077	− 0.297^**^	− 0.007	− 0.264^**^	1

*Correlation is significant at the 0.05 level (2-tailed)

**Correlation is significant at the 0.01 level (2-tailed)

**Table 4:** Correlation between happiness and quality of work life

	Total OHI	Adequate and fair compensation	Safe and healthy environment	Development of human capacities	Growth and security	Social integration	Constitutionalism	Total life space	Social relevance
Total OHI	1								
Adequate and fair compensation	0.128*	1							
Safe and healthy environment	0.170**	0.582**	1						
Development of human capacities	0.157**	0.231**	0.327**	1					
Growth and security	0.126*	0.249**	0.402**	0.335**	1				
Social integration	0.123*	0.184**	0.287**	0.413**	0.459**	1			
Constitutionalism	0.213**	0.330**	0.377**	0.427**	0.303**	0.444**	1		
Total life space	0.153*	0.355**	0.449**	0.397*	0.280**	0.270**	0.523**	1	
Social relevance	0.142*	0.326**	0.341**	0.377**	0.414**	0.349**	0.362**	0.413**	1

*Correlation is significant at the 0.05 level (2-tailed)

**Correlation is significant at the 0.01 level (2-tailed)

**Table 5:** Correlation between happiness and job satisfaction

	Total OHI	Satisfaction with knowledge	Satisfaction with diversity	Satisfaction with control	Satisfaction with salary
Total OHI	1				
Satisfaction with knowledge	0.146*	1			
Satisfaction with diversity	0.252**	0.373**	1		
Satisfaction with Closure	0.322**	0.203**	0.582**	1	
Satisfaction with salary	0.244**	0.156*	0.489**	0.600**	1

*Correlation is significant at the 0.05 level (2-tailed)

**Correlation is significant at the 0.01 level (2-tailed)

### Determinants of happiness

The results obtained from linear regression indicated that only economic status (B = 0.129, 95% CI = 0.435–8.584, P = 0.030) and ‘satisfaction with closure’ (B = 0.21, 95% CI = 0.29–2.07, P = 0.009) were significant contributing factors to happiness among nurses working in emergency departments (R^2^ = 0.38). Assumptions for multicollinearity were examined because of the combination of variables. The tolerance in the regression equation was less than 1.00, and the VIF in the final model was less than 2.50; thus, the assumptions for multicollinearity were not violated. The results are shown in Table 6.

**Table 6:** Determinants of happiness as obtained from multiple linear regressions analysis*

	Coefficients					Collinearity diagnostics	
	Unstandardized B	SE	Standardized B	95%CI for B	P value	Tolerance	Variance inflation factor
*Economic status*	4.510	2.069	0.129	0.435 to 8.584	0.030	0.952	1.640
*Quality of Work Life*							
Adequate and fair compensation	− 0.094	0.378	− 0.018	− 0.838 to 0.650	0.804	0.610	1.640
Safe and healthy environment	0.049	0.330	0.012	− 0.601 to 0.698	0.883	0.522	1.915
Development of human capacities	0.183	0.319	0.041	− 0.444 to 0.810	0.566	0.671	1.490
Growth and security	0.007	0.412	0.001	− 0.805 to 0.819	0.987	0.649	1.540
Social integration	− 0.057	0.370	− 0.011	− 0.785 to 0.671	0.877	0.632	1.582
Constitutionalism	0.640	0.402	0.125	− 0.152 to 1.432	0.113	0.539	1.854
Total life space	− 0.297	0.490	− 0.046	− 1.262 to 0.667	0.544	0.581	1.720
Social relevance	− 0.179	0.302	− 0.044	− 0.773 to 0.415	0.553	0.613	1.631
*Job Satisfaction*							
Satisfaction with Information	0.199	0.190	0.068	− 0.175 to 0.574	0.295	0 .806	1.241
Satisfaction with Variety	0.044	0.203	0.018	− 0.355 to 0.444	0.827	0.501	1.995
Satisfaction with Closure	1.187	0.451	0.217	0.298 to 2.076	0.009	0.492	2.034
Satisfaction with Pay	0.506	0.407	0.099	− 0.296 to 1.307	0.215	0.531	1.883

*All significant findings from correlation analyses were entered into stepwise regression analysis where happiness was treated as dependent variable. This table presents the final results

## Discussion

The findings indicated that overall nurses working in emergency departments felt a moderate level of happiness. Similarly, studies from Iran reported a moderate level of happiness among nursing students [29–31] or among hospital nurses [32].

However, there are also studies that reported Iranian nurses who worked in hospitals had low happiness [33, 34], confirming that hospital nurses have low to moderate happiness probably due to negative feelings they experience during patient care delivery, difficult work conditions, high workload, ineffective managerial policies, limited managerial support, unfair payments, equipment shortage, and limited career advancement opportunities [9].

Happiness is considered as the personal perspective about a favorable and pleasant state [2]. Individuals with more pleasant feelings are more satisfied with their job. In fact, happy individuals evaluate their skills and abilities very positively and remember positive events more frequently than negative ones. Therefore, they exchange positive energy with others and their environment, improve their relationships with them, and therefore, feel more satisfied with their job, colleagues, and workplace.

The findings also showed that the nurses did not have an optimum level of quality of working life. Nevertheless, similar findings were reported from Iran where studies found the low quality of working life for nurses [35, 36] suggesting that there might be the need to make some changes in a current health care environment [16, 37].

Job satisfaction is considered to be an important part of nursing since it directly or indirectly could affect patients’ care. We found that the study participants had a low level of job satisfaction. Not surprisingly similar findings were reported by other investigators from Iran [38–41] which we believe should be taken as a serious issue by health authorities. It is argued that job dissatisfaction usually occurs when there are problems with incivility at the individual, collective and organizational levels and might differ in West and East [42].

The study results indicated a significant correlation between the quality of working life and happiness whereas some other studies found no significant relationship between quality of working life and happiness but found a positive correlation between the quality of working life and job satisfaction, indicating that improvement in the quality of working life could have a positive impact on the overall job satisfaction [43]. However, we found that there is a significant association between job satisfaction and happiness indicating that the more happiness, the more job satisfaction [34, 44].

The results obtained showed that among independent variables that were entered into regression analysis only economic status, and ‘satisfaction with closure’ were the predictors of nurses' happiness.

It has been shown that those personal, work-related, and workplace-related factors were the most principal factors behind nurses' happiness [31]. Jun and Jo also found public sincere admiration of nursing, academic performance, physical health status, and the reasons behind entering nursing as the most significant factors contributing to nursing students' happiness [29]. Other predictors of nurses' happiness were economic status. Previous studies also reported a positive correlation between salary and happiness among different populations [45–47]. According to economic theory, living conditions, especially income have a lasting impression on happiness [48] and the results of various studies have confirmed this [49] Higher salary promotes nurses and their families’ welfare and therefore, eases their financial strain, helps them have an easier life, facilitates their task performance, and thereby, gives them a sense of happiness. Besides the amount of salary, satisfaction with salary was also a significant predictor of happiness among nursing personnel. Staff usually compares their own salaries with the salaries of other staff in or out of their organizations. Then, if they observe consistency between their own salaries and other staff's, they feel greater satisfaction with their salaries and greater happiness, and hence, will provide quality care. Moreover, consistency between workload and salary can contribute to their happiness [50].

The final factor behind nurses' happiness was their job satisfaction. This is in line with the findings of previous studies satisfaction with closure is the dimension of perceived job satisfaction, which determines how an employee perceives his/her job as a source of opportunity that provides him/her enough opportunity to complete the work from start to finish [31, 51–53].

## Limitations

This study had some limitations. The present study included only nurses working in the emergency departments in Ilam University of Medical Sciences and thus could not be generalized to all nurses. Additionally, although we performed linear regression analysis, the cross- sectional nature of the study should be considered when interpreting the results. Finally, one should note that performing such studies without any consideration for implementing specific interventions to improving the quality of working life among nurses working in emergency departments would not be advised given the importance of the finding in the current study. We recommend the future investigators take appropriate measures in this regard and contribute to increasing happiness among this population.

## Conclusion

Overall the current study found that nurses who work in emergency departments moderate levels of happiness. Additionally, the findings suggest that their happiness was associated with their economic status and closure over their duties.

## Data Availability

The datasets are available from the corresponding authors on request.

## Abbreviations

QWL: Quality of Work Life
JSQ: Job Satisfaction Questionnaire
OHI: Oxford Happiness Inventory
